# Supplementary figures and images for: Osteoporosis is associated with increased CVD mortality and all-cause mortality in alcohol-consuming individuals: A cohort study using data from NHANES
Source: PLoS One. 2025 Jun 26;20(6):e0327180. doi: 10.1371/journal.pone.0327180 (PMC12200671; doi:10.1371/journal.pone.0327180)

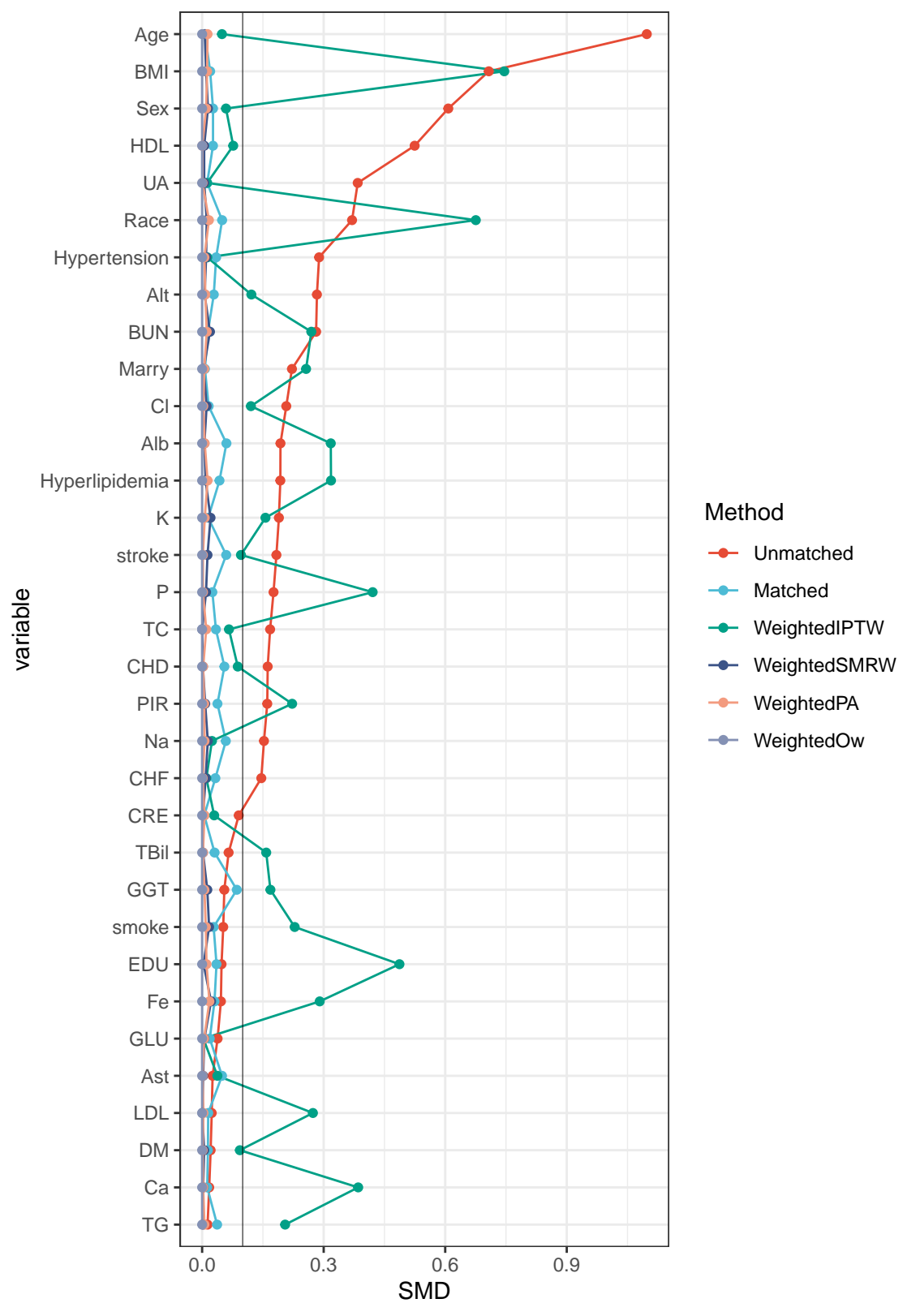

Supplement: S1 Fig — (PDF) [file pone.0327180.s001.pdf]
