# Supplementary material for: Osteoporosis is associated with increased CVD mortality and all-cause mortality in alcohol-consuming individuals: A cohort study using data from NHANES
Source: PLoS One. 2025 Jun 26;20(6):e0327180. doi: 10.1371/journal.pone.0327180 (PMC12200671; doi:10.1371/journal.pone.0327180)
Supplement: S1 Table — (DOCX) [file pone.0327180.s002.docx]

Supplementary Table 1 Association Between Osteoporosis and CVD and All-Cause Mortality After Propensity Score Matching.

|  | Model1 |  | Model1 |  | Model1 |  |
| --- | --- | --- | --- | --- | --- | --- |
|  | HR(95%CI) | P_value | HR(95%CI) | P_value | HR(95%CI) | P_value |
| **All-cause mortality** |  |  |  |  |  |  |
| OP- | Reference |  | Reference |  | Reference |  |
| OP+ | 1.29 (1.02~1.63) | 0.032 | 1.36 (1.07~1.72) | 0.01 | 1.32 (1.03~1.67) | 0.026 |
| **CVD mortality** |  |  |  |  |  |  |
| OP- | Reference |  | Reference |  | Reference |  |
| OP+ | 1.1 (0.71~1.73) | 0.667 | 1.18 (0.75~1.87) | 0.472 | 1.1 (0.68~1.79) | 0.701 |

Model 1: Adjustment

Model 2: adjusted for age (continuous), race and ethnicity (non Hispanic white, non Hispanic black, Mexican American, other Hispanics, other/multiracial), education level (lower than high school, high school graduation or equivalent, college graduation or above), smoking status (smokers, non-smokers), drinking status (drinkers, non-smokers) BMI (18.5-24.99 kg/m2, 25-29.99 kg/m2, ≥ 30 kg/m2), coronary heart disease, stroke, congestive heart failure, hyperlipidemia, hypertension, diabetes.

Model 3: Further adjust serum glucose, alanine aminotransferase, aspartate aminotransferase, total bilirubin, serum albumin, glutamine transpeptidase, serum creatinine, serum uric acid, blood urea nitrogen, serum sodium ions, serum phosphorus, serum calcium ions, serum potassium ions, serum iron ions, serum chloride ions, triglycerides, total cholesterol, high-density lipoprotein, and low-density lipoprotein based on Model 2.
